# Reframing Histological Risk Assessment of Oral Squamous Cell Carcinoma in the Era of UICC 8th Edition TNM Staging

**DOI:** 10.1007/s12105-020-01201-8

**Published:** 2020-07-13

**Authors:** Naomi Rahman, Morna MacNeill, William Wallace, Brendan Conn

**Affiliations:** 1 Edinburgh Dental Institute, Lauriston Building, Lauriston Place, Edinburgh, EH3 9HA Scotland; 2 Present Address: East Surrey Hospital, Canada Avenue, Redhill, RH1 5RH UK; 3 Royal Infirmary of Edinburgh, 51 Little France Crescent , Old Dalkeith Road, Edinburgh, EH16 4SA Scotland

**Keywords:** Oral squamous cell carcinoma Pattern of invasion, Lymphocytic host response, Perineural invasion

## Abstract

**Objectives:**

To assess whether application of the risk model originally proposed by Brandwein-Gensler, influences survival and disease progression in patients treated for oral squamous cell carcinoma (OSCCs)

**Materials and Methods:**

Tumours from 134 T1 and T2 OSCC resections (7th edition) were scored independently by 3 histopathologists according to worst pattern of invasion (WPOI), lymphocytic host response (LHR) and perineural invasion (PNI) and categorised according to risk score. Local recurrence, locoregional recurrence, disease progression and overall survival were study endpoints. Interobserver variability of pathologist scoring was also assessed.

**Results:**

Seventy-two patients (54%) were classified with low or intermediate risk and 62 (46%) patients were ‘high risk’. The inter-observer agreement was in moderate to strong agreement with the consensus scores (k range = 0.45–0.82). There was statistical significance between distant metastasis and ‘high risk’ tumours. Thirty tumours were upstaged to T3 in the 8th edition TNM staging, of which 83% had high risk scores. Overall risk score and TNM8 T stage has significant correlation with overall survival in comparison to the TNM 7 T stage.

**Conclusion:**

‘High risk’ tumours were significantly associated with distant metastasis possibly due to the greater likelihood of aggressive features such as WPOI and PNI. Primary tumours are more likely to express high risk features with increasing T stage. None of the patients classified as ‘low risk’ died perhaps suggesting these tumours represent a rare variant of OSCC with excellent prognosis.

## Introduction

Oral squamous cell carcinoma (OSCC) is the most common malignancy of the head and neck [1] with estimated global deaths from oral cancer of 177,000 in 2018 [2]. Despite advances in diagnostic techniques and treatment modalities, the overall survival of patients with head and neck cancer has not improved significantly over the past 20 years [3].

The evaluation of the clinical characteristics and anatomical extent of the tumour as well as its relationship to host tissues plays an important role in the prognosis of oral squamous cell carcinoma. Many studies stress importance of documenting the histological features in the pathology reports of resected oral cancer specimens [4]. In the UK, it is recommended that the information is gathered synoptically in the format of the RCPath dataset for histopathological reporting of mucosal malignancies of the oral cavity although the prognostic influence of each core data item, despite being supported by best available evidence, is unclear on a quantitative basis and difficult to apply with absolute confidence when deciding on treatment modalities in multidisciplinary meetings.

Many histological grading systems have been described, however there is a lack of agreement as to a satisfactory robust predictive model. The Broders grading system, established in 1920 and still recommended by WHO [5], [6] is probably the best known attempt at prognostication with subjective assessment of key histological features (degree of differentiation, cellular pleomorphism and mitotic activity) and graded as well, moderately or poorly differentiated. This system is well recognised and universally adopted however is of questionable discriminatory value given that up to 90% of oral tumours may be moderately differentiated [6]. Annoreth et al. focused on the relationship between the tumour and surrounding tissue [7] including parameters such as the degree of pattern of invasion, stage of invasion and leukocyte infiltration. Bryne et al. later developed a system of invasive front grading (IFG) with 5 histological features including host response [8]. These models have not been successfully due to small sample sizes, heterogeneous tumour sites and evaluation of different specimen types (biopsies only and resection specimens only) [9].

More recently, studies by Woolgar et al. have further explored prognosticators for local regional recurrence, lymph node metastasis and distant metastasis [6–8]. In 1998, the UK RCPath developed detailed guidelines together with the standard minimal dataset proforma [10], [11] many of the core data parameters are supported by the evidence from studies conducted by Woolgar.

**Fig. 1:**
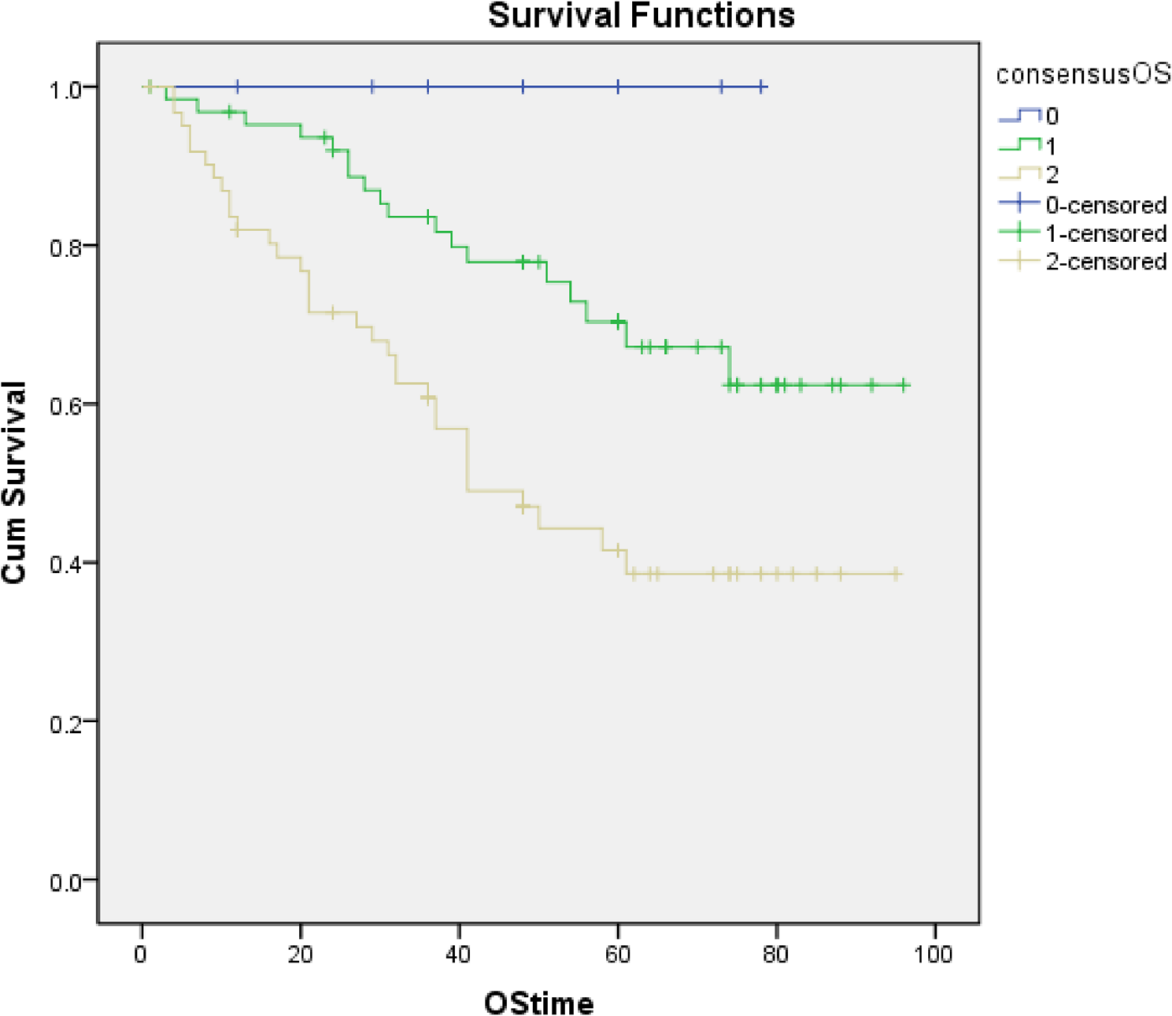
Kaplan Meier curve to show the risk score against overall survival (Risk 1 = low, Risk 2 = intermediate Risk 3 = high risk)

In 2005, Brandwein-Gensler and co-workers introduced a histological risk assessment model purported to having superior prognostic value in comparison to previously described systems [9]. The model is based on the cumulative evaluation of 3 key histological parameters: Worse pattern of invasion (WPOI), lymphocytic host response (LHR) and perineural invasion (PNI) as shown in Fig. 1. All 3 parameters are scored and the total points from the 3 variables are added. If the total score = 0 this is considered to be low risk, if the score is one or 2, this is considered intermediate risk and if the score is greater than 3, this is categorised as high risk (Table 1) [9].

**Table 1:** Brandwein Gensler’s Validated Histological Risk Model

Variable	Definition	Point assignment
WPOI		
Type 1	Pushing border	0
Type 2	Finger-like growth	0
Type 3	Large separate islands, more than 15 cells per island	0
Type 4	Small tumour islands, 15 cells or fewer, per island	+1
Type 5	Tumour satellites, ≥1 mm from main tumour or next closest satellite	+3
LHR		
Type 1	Dense complete host response rimming tumour	0
Strong	Lymphoid nodules at advancing edge in each 4x field	
Type 2	Intermediate host response	+1
Intermediate	Lymphoid nodules in some but not all 4x fields	
Type 3	Little or no host response	+3
Weak	No lymphoid nodule	
PNI		
None	None	0
Small nerves	Tumour wrapping around nerves, <1 mm diameter	+1
Large	Tumour wrapping around nerves, equal to or greater than 1 mm diameter (20+)	+3

Li et al. [12]

The risk model has been shown to correlate significantly with local regional recurrence (*p* = 0.0004) and overall survival (*p* = 0.0001); particularly in low stage oral carcinomas. The model places weighted point values on more aggressive features [12]. It is suggested that high-risk low stage oral carcinomas may benefit from adjuvant radiotherapy; even in the case of satisfactory margins.

The 8th edition of the TNM staging for oral squamous cell carcinoma, published in 2017, places emphasis on depth of invasion as well as maximum diameter in determining T stage [13] and represents somewhat of a paradigm shift in oral and maxillofacial pathology. A combined assessment of histological grading as well as clinical staging may be a better and more accurate way of predicting the outcome of the neoplasm and deciding the best treatment for each patient [1].

The primary aim of this study is to assess whether the morphological signatures of the risk model have influence over disease progression in cases of oral squamous cell carcinoma. A secondary aim was to investigate the influence that the recent changes to T staging in the UICC/AJCC TNM 8th edition may have on the histological risk model.

## Materials and Methods

The institutional review for human subject research reviewed and approved this study. One hundred and thirty-four patients with oral squamous cell carcinoma treated with primary resection between 2009 and 2014 were included in the study to allow a minimum follow-up time of 5 years. The pathology reports were reviewed retrospectively and target cases were identified by applying the inclusion and exclusion criteria. The inclusion criteria were: complete demographic and clinical data, T1 T2 (7th edition) OSCC treated with surgery with or without post-operative oncology therapy, availability of slides, paraffin embedded blocks and follow-up data of at least 5 years for survival. The exclusion criteria were as follows: T3 & T4 primary tumours, recurrences, secondary tumours, oropharyngeal tumours including tongue base and tonsil, depth of invasion of less than 1 mm, non-conventional squamous cell carcinoma, squamous carcinoma with prominent intraductal component and patients seropositive for HIV. Cases with positive lymph node metastasis in accompanying neck dissections were included. All pathology slides from the resection specimens were retrieved and reviewed. All H&E stained slides from tumour resections were independently reviewed by 3 pathologists blinded to the demographic data and outcomes. The slides were scored according to the 3 components of the risk model: Worst pattern of invasion (WPOI), lymphocytic host response (LHR) and perineural invasion (PNI) and then categorised according to the total combined score. Individual analyses by consultant pathologists were verified by a consensus meeting at multi-headed microscope. RC Path minimum dataset items were transcribed from the original pathology report. Age, gender, date of surgery, TNM stage was collected on Microsoft access databases. For patients treated with accompanying neck dissection (*n* = 85) the presence or absence of lymph node metastasis was recorded along with the presence and absence of extracapsular spread. Ethics approval and access to histology slides and clinical data was approved from the local tissue governance team (SR679). The cases were selected consecutively with unknown outcomes.

The endpoints assessed were local recurrence (LR), regional recurrence (RR), distant recurrence (DR) and recurrence free survival (RFS).

Cross tabulations, Mann Whitney tests and Chi squared tests were used to assess correlations between histopathological risk model, prognosticators in the RCpath dataset and outcomes. Continuous data was summarised using means and ranges (minimum and maximum). Categorical data by using frequency counts and percentages. Inter observer variability was assessed using kappa statistics. Oncological outcomes were expressed using the Kaplan-Meier method. Univariate analysis of outcome was performed using the Log-Rank test and multivariate analysis using the Cox regression model. Hazard ratios were calculated using a univariate Cox regression model, with 95% confidence intervals. Results were considered significant at the *p* < 0.05 level. All calculations were done using SPSS.

## Results

**Fig. 2:**
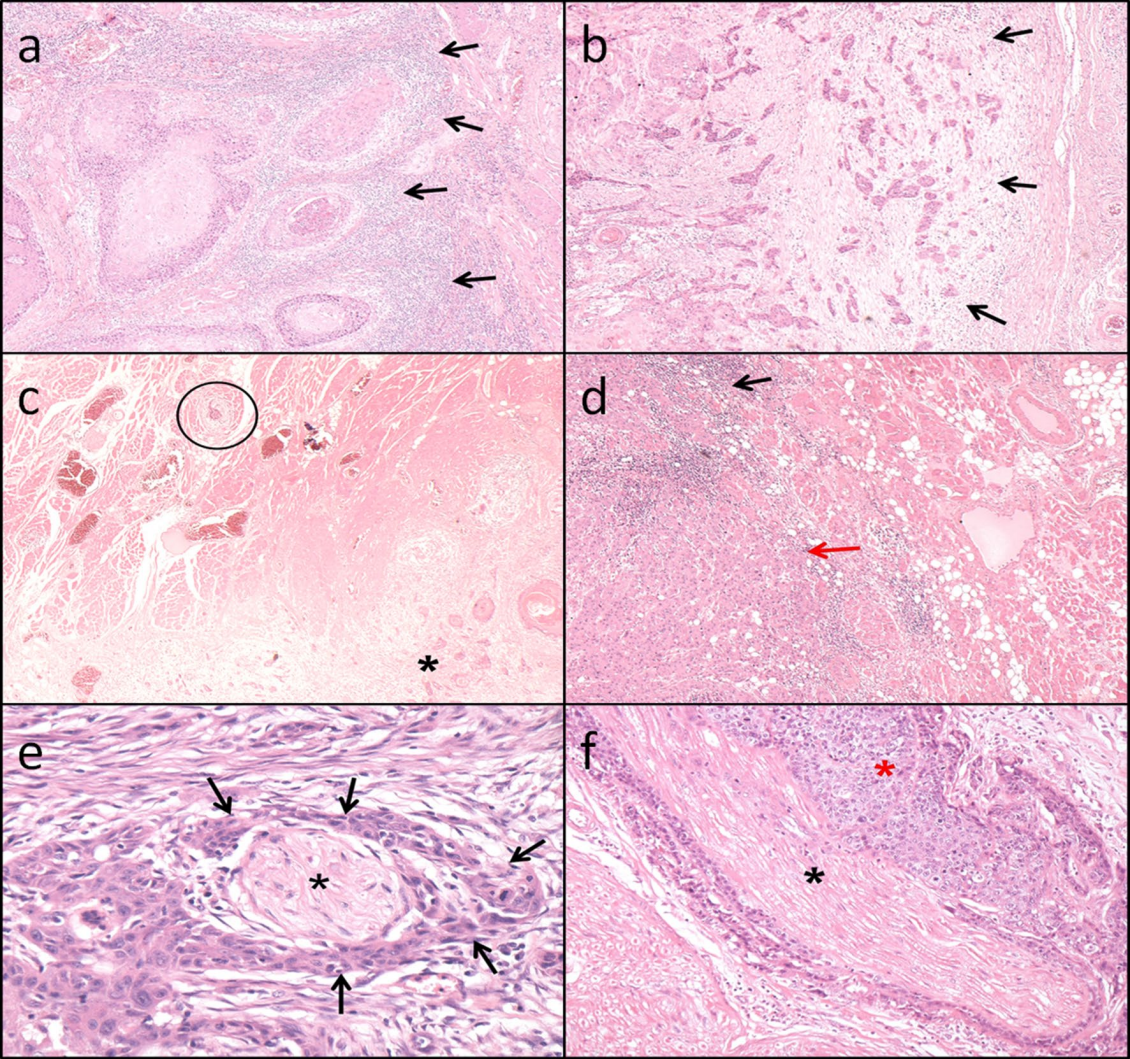
(**a**) H&E × 40. Oral squamous cell carcinoma showing invasion in the form of discrete large islands consisting >15cells (WPOI3) and a strong continuous lymphoid response around the periphery of the whole tumour (black arrows). In the absence of perineural invasion; this is classified as a low risk tumour (risk score 0). (**b**) H&E × 40. Oral squamous cell carcinoma showing invasion in the form of discrete small islands and groups comprising less than 15 cells (WPOI4) featuring a barely discernible lymphoid host response at the invasive front (black arrows). Even in the absence of PNI the total risk score would be at least 4 with the tumour classified as high risk. (**c**) H&E × 20. Oral squamous cell carcinoma showing widely dispersed invasion with a single small group of malignant cells (black circle) situated more than 1 mm ahead of the main tumour (black star). This pattern of invasion equates to WPOI5 and carries a risk score of 3; rendering the tumour as tumour high risk regardless of LHR and PNI status. (**d**) H&E × 40. Oral squamous cell carcinoma showing an LHR of variable density with stronger (black arrow) and weaker zones (red arrow). This incomplete distribution of lymphoid cells at the invasive front is considered intermediate (risk score 1). (**e**) H&E × 200. Oral squamous cell carcinoma showing perineural invasion of a small nerve of <1 mm (black star). The tumour (black arrows) shows complete cirumferential invasion of the nerve. This finding carries a risk score of 1 and would require combination with an unfavourable pattern of invasion or lymphoid host response in order to qualify as a high risk tumour. (**f**) H&E × 200. Oral squamous cell carcinoma showing obvious perineural invasion of a large nerve >1 mm diameter (black star). The tumour (red star) shows almost complete circumferential invasion of the nerve. This finding carries a risk score of 3 and immediately qualifies a tumour as high risk regardless of WPOI or LHR status

The study group comprised 134 patients with primary T1 (*n* = 82, 61%) and T2 (*n* = 52, 39%) oral squamous cell carcinoma (TNM7). This consisted of 83 male and 51 female patients, aged between 22 and 88 years (mean age 63.4 SD 13.2). The clinical and demographic data are shown in Table 2. The follow-up ranged from 1 to 96 months with a mean of 53.5 months (SD 39.6). Eighty-three patients were recorded as still being alive while 51 patients had died giving an overall survival of 62%. Forty-three patients were reported to have disease progression and 91 were free of disease at last follow-up fragment. Eight patients had died of disease within 50 months. Twelve patients had local recurrence, 42 (31%) had locoregional recurrence of which 20 were classified as high risk according to the risk model but due to the small numbers there was no statistical significance found. Seven patients had distant metastasis all of which were reported as high-risk (*p* = 0.01). Eight patients had died of the disease with 6 recorded as high-risk (75%). Kaplan Meier curve (Fig. 2) shows the risk score against overall survival (HR 2.77 95% CI 1.56, 4.94, *p* = 0.001).

**Table 2:** Demographic data and T stage of OSCC patients. The table also shows the frequency distributions for each parameter: pattern of invasion, perineural invasion, lymphocytic host response and overall risk score for each tumour stage

Number of patients		134	
Male		83 (52%)	
Female		51 (38%)	
Age range		22–88	
Mean age (SD)		63.4 (13.2)	
Oral cavity			
T1		82 (61%)	
T2		52 (39%)	

Histological Characteristic	Tumour stage		Statistical significance
			(Mann-Whitney test)
	T1 (n = 82)	T2 (n = 52)	
Pattern of invasion			
WPOI1	1 (1%)*	0	
WPOI2	4 (5%)*	2 (4%)	*U* = 1591
WPOI3	25 (30%)*	8 (15%)	*n* = 134
WPOI4	43 (52%)*	28 (54%)	*p* = 0.07
WPOI5	9 (11%)*	14 (27%)	
Lymphocytic host response			
Strong	14 (17%)	5 (10%)	*U* = 1993.5
Intermediate	57 (70%)	40 (77%)	n = 134
Limited	11 (13%)	7 (13%)	*p* = 0.42
Perineural spread			
None	65 (81%)	25 (54%)	*U* = 1409
Small nerves	15 (14%)	17 (33%)	n = 134
Large nerves	2 (5%)	10 (13%)	*p* = 0.0001
Risk category			
Low	5 (6%)	3 (6%)	*U* = 1565
Intermediate	48 (59%)	16 (31%)	n = 134
High	29 (35%)	33 (63%)	*p* = 0.004

*Percentages do not add up to 100% due to rounding of numbers

None of the patients who died with or without disease had low risk score. High risk score (*p* = 0.03) and increasing T stage in TNM 8th edition (*p* = 0.054) had significant inverse correlation with overall survival. Seventh edition T stage had no such correlation.

Table 3 shows the frequency distributions for each parameter: pattern of invasion, perineural invasion, lymphocytic host response and overall risk score for each tumour stage. A Mann Whitney test showed that perineural invasion and high overall risk score were significantly greater in T2 tumours compared to T1 tumours (*p* = 0.004). The overall size and depth of invasion were recalibrated to satisfy the criteria for TNM 8 staging. Thirty-four tumours previously designated as T1 would now be classified as T2 and 30 T2 tumours would now be classified as T3 according to TNM 8th edition. A Mann Whitney test showed that the worst pattern of invasion (*p* = 0.01), perineural invasion (*p* = 0.0001) and the overall risk score (p = 0.0001) were significantly greater in the tumours reclassified as T3 by depth of invasion.

**Table 3:** T1, T2, T3 tumours according to the 7th and 8th edition of TNM staging and the distribution of the 3 parameters and overall score categories of the Risk Model

Histological Characteristic	Tumour Stage		Statistical significance	Tumour Stage			Statistical significance
	TNM 7th Edition		(Mann-Whitney test)	TNM 8th edition			(Mann-Whitney test)
	T1 (n = 82)	T2 (n = 52)		T1 (*n* = 48)	T2 (56)	T3 (*n* = 30)	
Pattern of invasion							
WPOI1	1 (1%)*	0	*U* = 1591	1 (2%)	0	0	*U* = 977
WPOI2	4 (5%)*	2 (4%)		4 (8%)	2 (4%)	0	
WPOI3	25 (30%)*	8 (15%)	n = 134	17 (35%)	11 (20%)	5 (17%)	n = 134
WPOI4	43 (52%)*	28 (54%)	*p* = 0.07	22 (46%)	36 (20%)	13 (43%)	*p* = 0.01
WPOI5	9 (11%)*	14 (27%)		4 (8%)	7 (13%)	12 (40%)	
Lymphocytic host response							
Strong	14 (17%)	5 (10%)	*U* = 1993.5	6 (13%)	10 (18%)	3 (10%)	*U* = 1417
Intermediate	57 (70%)	40 (77%)	n = 134	37 (77%)	38 (68%)	22 (73%)	n = 134
Limited	11 (13%)	7 (13%)	*p* = 0.42	5 (10%)	8 (14%)	5 (17%)	*p* = 0.38
Perineural spread							
None	65 (81%)	25 (54%)	*U* = 1409	42 (88%)	39 (70%)	9 (30%)	*U* = 714.5
Small nerves	15 (14%)	17 (33%)	n = 134	6 (13%)	15 (27%)	11 (37%)	n = 134
Large nerves	2 (5%)	10 (13%)	*p* = 0.0001	0	2 (4%)	10 (33%)	*p* = 0.0001
Risk category							
Low	5 (6%)	3 (6%)	*U* = 1565	4 (8%)	2 (4%)	2 (7%)	*U* = 849.5
Intermediate	48 (59%)	16 (31%)	n = 134	31 (65%)	30 (54%)	3 (10%)	n = 134
High	29 (35%)	33 (63%)	*p* = 0.004	13 (27%)	24 (43%)	25 (83%)	*p* = 0.0001

Risk scoring did not demonstrate an association between disease progression and the overall score or any of the 3 individual parameters as shown in Table 4. The Chi squared tests did not show a significant association between disease progression and the prognostic features recorded in the dataset (Table 5).

**Table 4:** Frequency distributions for free of disease and disease progression according to each parameter: pattern of invasion, perineural invasion, lymphocytic host response and overall risk score for each tumour stage

Histological characteristic	Status at follow up		
	Disease free	Disease progression	Statistical significance
	*n* = 92	*n* = 42	(Mann-Whitney U test)
Pattern of invasion			
WPOI1	1 (1%)*	0	
WPOI2	2 (2%)*	4 (10%)	*U* = 1797
WPOI3	27 (29%)*	6 (14%)	n = 134
WPOI4	47 (51%)*	24 (57%)	*p* = 0.48
WPOI5	15 (16%)*	8 (19%)	
Lymphocytic host response			
Strong	12 (13%)	7 (17%)	*U* = 18,357
Intermediate	70 (76%)	27 (64%)	n = 134
Limited	10 (11%)	8 (19%)	*p* = 0.65
Perineural spread			
None	63 (68%)	27 (64%)	*U* = 1831
Small nerves	22 (24%)	10 (24%)	n = 134
Large nerves	7 (8%)	5 (12%)	*p* = 0.56
Risk category			
Low	6 (7%)	2 (5%)	*U* = 1752
Intermediate	46 (50%)	18 (43%)	n = 134
High	40 (43%)	22 (52%)	*p* = 0.33

**Table 5:** Univariate analysis of the Risk Model and disease outcomes

Risk model	Total	Local recurrence		Regional recurrence		Distant metastasis		Deaths of disease	
		(% of total)		(% of total)		(% of total)		(% of total)	
	134	Yes	No	Yes	No	Yes	No	Yes	No
Low & Intermediate Risk scores	72 (54%)	5 (7%)	67 (93%)	15 (21%)	57 (79%)	0 (0%)	72 (100%)	2 (3%)	70 (97%)
High	62 (46%)	7 (11%)	55 (89%)	14 (23%)	48 (77%)	7 (11%)	55 (89%)	6 (10%)	56 (90%)
Total	134	12 (9%)	122 (91%)	29 (22%)	105 (78%)	7 (5%)	127 (95%)	8 (6%)	126 (94%)
Statistical Significance Chi-squared test		*p* = 0.38		*p* = 0.8		*p* = 0.03		*p* = 0.9	

The inter-observer agreement among the 3 pathologists was moderate to strong when measured against the consensus score (k range = 0.45–0.82). However, the scores showed weak to moderate agreement in WPOI, LHR, PNI and overall scores when comparing the pathologist score against each other as appears.

A spearman’s rank-order correlation (Table 6) was carried out to determine the relationship between the WPOI scores and the RCPath dataset classification of pattern of invasion. There was a strong positive correlation between noncohesive pattern and the more aggressive WPOI scores which was statistically significant (rs = 0.325, *p* = 0.0001).

**Table 6:** Spearman rank correlation between the 5 categories of WPOI described in Brandwein Gensler’s study and cohesive, mixed and non-cohesive patterns of invasion described in the Royal College of Pathologist dataset

	Cohesive	Non-cohesive	Mixed	*P* value
	*n* = 35	*n* = 71	*n* = 28	
WPOI1	0	1	0	0.0001
WPOI2	4	2	0	
WPOI3	18	8	7	
WPOI4	10	48	13	
WPOI5	3	12	8	

## Discussion

In the UK, multidisciplinary treatment decisions for OSCC patients are based primarily on stage and performance status with histological findings in post-operative resection specimens taken into account. There is a lack of agreement as to a predictive model and essentially all tumours are treated identically despite compelling evidence of morphological and behavioural diversity [9]. Conventional thinking dictates that as tumours get larger; they are more likely to express aggressive histological features as demonstrated in the original study by Margaret Brandwein-Gensler [15]. As staging is a robust predictor of prognosis in late stage disease, histological risk scoring is therefore considered to be of greatest practical value in low stage disease in attempt to identify smaller tumours that do inexplicably badly even if completely excised, perhaps as a result of unfavourable histological features. The 2013 Margaret Brandwein-Gensler study on low stage disease showed 20% of patients with high risk early stage SCC demonstrated locoregional recurrence (56/294) and 7% had shorter disease specific survival (18/294) [9]. WPOI5 in particular was identified as a significant concern in low stage disease with 42% of tumours exhibiting locoregional recurrence. Further studies have shown high-risk patients less than 60 years old with increased risk of recurrence (*p* = 0.022, HR11.2, 95% CI 1.4, 87.1) [14] and significant correlation with disease specific survival, disease free survival and overall survival [15]. In contrast, other studies have shown no significant correlation between epidemiological and clinical parameters and outcomes in relation to the histopathological risk model [16]. Further studies also found no significant correlation between risk model and disease progression or outcomes with the exception of gender (*p* < 0.0001) [1]. Critics of the risk model suggest poor discriminatory value of WPOI assessment in that noncohesive pattern of invasion, particularly in WPOI4, occurs in most SCC patients and therefore the value in predicting tumour behaviour is limited [16]. Indeed, WPOI4 was noted in 53% of our study population. It has also been suggested that lymphocytic response, while potentially eradicating tumour cells, can also aid tumourigenesis by production of several growth promoting signalling molecules (EC GF, VEGF, FGF2, 2, chemokines and cytokines). Hence the immune and inflammatory response may have both tumour promoting and anti-tumour effects. Therefore, dividing LHR into favourable and unfavourable types on histology without knowledge of the composition of the infiltrate may be potentially misleading. The precise composition of the lymphocytic host response is not investigated here (nor in other studies).

This risk model requires detailed analysis and cumulative scoring of 3 parameters which some may find burdensome in routine practice. Indeed, recent work from 2 groups have shown compelling prognostic value with detailed assessment pattern of invasion, or ‘tumour budding’ alone. [17, 18, 19] Tumours are divided into well differentiated (G1), Moderately differentiated (GII) and poorly differentiated (GIII) categories depending on the degree of budding alone [17] or in combination with degree of differentiation [19]. Boxberg et al. were able to demonstrate significantly reduced overall survival with increasing grade [17] and were furthermore able to demonstrate good inter and intra observer variability, particularly with training of individual assessors [18]. Elseragy et al. showed significant association with degree of budding and disease specific survival and good predictive value for grade and disease free survival in comparison with WHO recommended method of grading [19]. Both groups justifiably advocate incorporating tumour budding into future WHO grading schemes.

While assessment of pattern of invasion alone appears valuable in this context, we feel that the risk model scoring: taking perineural spread and lymphocytic host response into account along with pattern of invasion, allows a more holistic assessment of tumour in its environment. The technique described by Boxberg et al. is neatly described but may be potentially time consuming in practice, with a requirement for detailed assessment and scoring of 10 high power fields for each case. The technique of Elsegary et al. relies on a combination of tumour budding with differentiation which does not seem to allow complete separation from the current WHO assessment of differentiation. Indeed both techniques employ existing terminology (well, moderately and poorly differentiated) which may lead to confusion when transitioning to an updated grading scheme. Importantly, the degree of budding in both studies would be, by definition, no more than WPOI4 according to the risk model. Both methods lack a WPOI5 equivalent which has been shown by other workers to have the greatest predictive value in term of local regional recurrence [12].

Our study showed a significant association with risk score and increasing tumour size in which T2 (7th edition) were statistically more likely to demonstrate perineural invasion (*p* = 0001) and high risk score (*p* = 0.004) than T1. This was further echoed in application of TNM8 criteria in which WPOI (*p* = 0.0001), PNI (p = 0.0001) and high risk score (p = 0001) were shown to be more likely in tumours upstaged according to depth of invasion. This finding lends histological justification to the addition of assessment of depth of invasion in staging. Furthermore, our study demonstrated a significant association with high-risk score and both overall survival (*p* = 0.03) and distant metastasis (p = 0.0001).

Whilst our study showed a greater proportion of high-risk tumours with disease progression compared to intermediate and low risk types, the 3 parameters of the risk model and the overall score did not have a statistically significant association. This is potentially due to a relatively small sample size and a low event rate.

Despite the clear histological differences demonstrated between T1 & T2 tumours here, a somewhat unusual finding was that a greater percentage of T1 tumours (7th edition) in this study population showed disease progression (36.5%) in comparison to T2 (25%). It was considered that T2 tumours were perhaps subject to more extensive primary surgery including a neck dissection and were more likely to have post-operative oncological treatment. A proportion of T1 tumours may have also been identified histologically in more conservative excision specimens of suspicious oral dysplastic lesions. Indeed 71% of T2 tumours were excised with clear peripheral margins > 5 mm and 71% were excised with clear deep margins > 5 mm. By contrast, only 48% of T1 patients had clear peripheral margins > 5 mm while 62% had clear deep margins > 5 mm. An important observation here is that 7th edition T stage was not a good indicator of prognosis. Application of TNM8 showed significant association with overall survival here but further studies are needed.

One of the aims of the study was to identify the ease in introducing the histological risk model to routine pathology practice. In our experience, the risk model approach encouraged closer more objective analysis of the tumour and can be easily incorporated into a synoptic report. Consensus meetings were especially helpful. However, it is noted that application of the criteria may be time consuming and difficulties may be readily encountered in deciding between certain WPOI types (particularly WPOI 4& 5 and WPOI 2&3). We noted that there is large variation/heterogeneity of LHR within individual tumours and agree with previous investigators that it is necessary for the whole host-tumour interface to be analysed for precise scoring. The technique is therefore not applicable to incisional biopsies, however the finding of WPOI5 and PNI (particularly in large nerves) in an incisional biopsy may be used to communicate the likelihood of uncovering a high risk phenotype on resection to aid surgical planning. Indeed, while not statistically significant, disease progression was noted to have occurred in 50% of patients with PNI of large nerves, 38% of WPOI5 cases and 37% of patients with high risk score.

The interobserver agreement between pathologists was moderate to strong when assessed against the consensus scores (k range = 0.45–0.82). In the consensus meeting, our pathologists realised that it can be difficult to distinguish between criteria WPOI4 and WPOI5 particularly if the pathologist fails to identify a subtle group of tumour cells 1 mm ahead of the main mass. Difficulty was also encountered in separating WPOI3 from WPOI2 especially if finger like projections cut at an angle potentially appeared as discrete islands. It was further noted that assessment of the different patterns of LHR is largely subjective in contrast to perineural invasion which can be assessed objectively. Most difficulties were easily resolved at the multihead microscope with all present. In daily practice, difficulties and inconsistencies in risk scoring could be overcome by double reporting with a pathologist colleague with training in risk scoring, review of cases by a second pathologist prior to presentation of the case at MDM and regular audit.

## Conclusion

This study highlights both benefits and limitations of using the histopathological risk score in practice. The risk score is significantly associated with overall survival and distant metastasis. There was a non-significant association with disease specific mortality (75%) and local and regional recurrence in high-risk tumours. In our study, we found that all 30 tumours which would now be classified as T3 tumours in the 8th edition TNM were significantly more likely to exhibit features amounting to high risk score adding justification to the recent changes. TNM8 T stage had better correlation with overall survival than TNM7. In the authors experience, although the risk scoring can be difficult technically, agreement between pathologists improved with consensus. Differentiating between categories WPOI4 and WPOI5 allows further substratification within tumours exhibiting a non-cohesive growth pattern. There was a significant correlation between WPOI and RCPath POI criteria and therefore it could be feasible to score pattern of invasion in more detail using the risk model WPOI criteria. Interestingly, none of the 8 patients with a risk score of zero died. This may indicate low risk OSCC is a rare subtype with excellent prognosis. Further study is needed.

## Abbreviations

OSCC: Oral squamous cell carcinoma
POI: Pattern of invasion
WPOI: Worst pattern of invasion
TIL: Tumour infiltrate lymphocyte
LHR: Lymphocytic host response
PNI: Perineural invasion
